# Polyoxyethylene Diamine Modification of Poly(amide-imide)-polyethylene Glycol Exhibits Excellent Hydrophilicity, Degradability, and Biocompatibility

**DOI:** 10.3390/polym14214694

**Published:** 2022-11-03

**Authors:** Ran Yu, Chao Xu, Xiaopei Wu, Honglian Dai

**Affiliations:** 1 Foshan Xianhu Laboratory of the Advanced Energy Science and Technology Guangdong Laboratory, Xianhu Hydrogen Valley, Foshan 528200, China; 2 State Key Laboratory of Advanced Technology for Materials Synthesis and Processing, Wuhan University of Technology, Wuhan 430070, China

**Keywords:** polyoxyethylene diamine (H_2_N-PEG-NH_2_), poly(amide-imide) (PAI), biocompatibility

## Abstract

We designed and synthesized the polyoxyethylene diamine (H_2_N-PEG-NH_2_) and poly(amide-imide)-polyethylene glycol (PAI-PEG) copolymers. The physical and chemical properties, mechanical properties, and in vitro biocompatibility of the materials were characterized. The results showed that the best elongation at break and recovery were obtained when the amount of PEG was 5 wt%. With the increase in PEG content, the degradation rate, hydrophilic property, tensile strength and tensile modulus of the copolymer decreased to a certain extent. The material had the best thermal stability and mechanical properties when 5 wt% PEG was added. Cytocompatibility evaluation showed that the addition of PEG could enhance the cell compatibility of the material and make it potentially suitable for application in bone repair.

## 1. Introduction

In the United States and the European Union, there are approximately 1 million cases of bone defects each year, and bone graft surgery is required to achieve fusion [1]. Bone is a dynamic, highly vascularized tissue with a unique ability to heal and reshape without leaving scars [2,3]. Therefore, materials are booming in the medical field of artificial bones, and among them, synthetic polymers have attracted more and more attention due to their designable properties [4,5,6]. Although polymer materials have been widely used in the field of bone repair, artificial polymer materials still have disadvantages that cannot be ignored, such as non-degradability, stress shielding and lack of biological activity. Therefore, the ideal bone repair material should have good biocompatibility, biodegradability and suitable mechanical properties.

Poly(amide-imide) (PAI) was chosen in our work because it has been widely used in the industrial field as an engineering plastic due to its excellent mechanical properties and extremely high thermal stability. However, its lack of biodegradability limits its application in the field of biomaterials. Previous studies have introduced L-amino acids into non-degradable polymers [7,8,9] to make the non-degradable materials biodegradable. The reason is that amino acids can endow the polymer materials with bioactive sites similar to peptide materials, and also provide enzymatic hydrolysis sites for the biodegradation of polymers [10,11,12]. In our previous research [13], the synthesized PAI derived from L-phenylalanine showed good biocompatibility, biodegradability, and potential application value in the field of biological materials. However, the hydrophilicity of PAI is poor. Therefore, we hope to improve the hydrophilicity of PAI by introducing the hydrophilic polyether segment to form copolymers with PAI.

Polyethylene glycol (PEG) is a polymer widely used in the field of biomaterials with good hydrophilicity. In an aqueous medium, the molecular conformation of PEG will change, and the ether bond (R-O-R) in PEG will be distributed on the chain surface, making it easy to combine with water around the polymer [14]. Its main applications in biomaterials include increasing membrane permeability and modifying some materials with poor biocompatibility. PEG with higher molecular weight can be cleared by the kidney, so it has safe toxicity characteristics and bio-tolerance. Due to the wide range of PEG molecular weight selection, the range of mechanical and physical properties can be realized by simply changing the molecular weight and concentration of PEG, and biological activity can be controlled by introducing specific biological active agents [15]. The activity of the terminal hydroxyl in polyethylene glycol is relatively low, and a large number of studies have modified the terminal hydroxyl into more reactive functional groups such as amino (-NH_2_), carboxyl (-COOH), and ester groups [16,17]. The modified PEG can be used to further synthesize new polymer materials. With the development of material technology, in the field of medical materials the single function of a material cannot meet its application requirements, so more and more studies have been devoted to the preparation of composite polymers and copolymers.

PAI can provide good mechanical properties and thermal stability for the copolymer, while PEG can provide excellent hydrophilicity and biocompatibility. To make up for the lack of hydrophilicity of PAI, PEG was copolymerized with PAI. It was hoped that PAI-PEG copolymer could have good hydrophilicity, biocompatibility, and suitable mechanical properties. In our work, PAI-PEG copolymers were prepared by chemical bonding. Finally, we tested the cell compatibility of the copolymer to evaluate its potential as a bone repair material.

## 2. Materials and Methods

### 2.1. Materials

The following materials were used: polyethylene glycol (Mn = 2000), dichloromethane (AR, ≥99.5%), triethylamine (AR, 99%), p-toluene sulfonyl chloride (TsCl; ≥99%), ethyl ether (AR, ≥99%), L-phenylalanine (99%), glacial acetic acid (AR, ≥99.5%), anhydrous ethanol (AR, ≥99.7%), hydrochloric acid (AR, 36~38%), sodium bicarbonate (NaHCO_3_; AR, ≥99.5%), sodium hydroxide (NaOH; AR, ≥96%), calcium nitrate tetrahydrate (AR, 99%), diammonium phosphate (AR, ≥99%), ethyl orthosilicate (TEOS; AR), N, N-dimethyl acetamide (10-channel DMAc; AR, ≥99%), and ammonia water (AR, 25~28%) were purchased from Sinopharm Chemical ReagentCo., Ltd. (Shanghai, China). 3,3′,4,4′-biphenyl tetracarboxylic acid dianhydride (BPDA; 99%), 4,4′-two carboxyl diphenyl ether (ODA; 98%), tetrabutylammonium bromide (TBAB; AR, 99%), triphenyl phosphite (TPP; 98%) and tetrabutyl ammonium bromide (TBAB; AR, 99%) were purchased from Shanghai Aladdin Biochemical Technology Co., Ltd. (Shanghai, China). Mouse bone marrow mesenchymal stem cells (BMSCs) were extracted from the bone marrow of male Sprague-Dawley (SD) rats (Hubei Provincial Center for Disease Control and Prevention, Wuhan, China). alpha MEM medium, 10% Fetal Bovine Serum (FBS), penicillin-streptomycin, phosphate buffer saline (PBS), and trypsin-edta were purchased from Hyclone (Logan, UT, USA). Additionally, we used Cell Counting Kit-8 (CCK-8; Beyotime Biotechnology., Wuhan, China), fitc-phalloidin (Solarbio, Beijing, China), 4′,6-diamidine-2-phenylindoles (DAPI; Beijing solarbio science﹠ technology Co., Ltd., Beijing, China), and 4% paraformaldehyde (Wuhan Boster Biological Technology., LTD, Wuhan, China).

### 2.2. Synthesis of Polyoxyethylene Diamine (H_2_N-PEG-NH_2_)

Polyethylene glycol (PEG, molecular weight 2000, PEG 2000) and p-toluene sulfonyl chloride (TsCl) were dissolved in dichloromethane (CH_2_Cl_2_) and triethylamine to form a solution. The reaction was terminated after stirring the solution for 12 h at room temperature, then the organic phase solution was washed with HCl (3M). The solid sodium bicarbonate was then slowly added to the organic phase to react with the HCl away. The solution was stirred vigorously until there were no bubbles, then filtered under reduced pressure to produce a concentrated solution. Excess diethyl ether was slowly added to the concentrated solution to precipitate a large amount of sediment. After filtration and vacuum drying, the white waxy solid product polyethylene glycol p-toluene sulfonate (TsO-PEG-OTs) was obtained.

**Figure 1:**
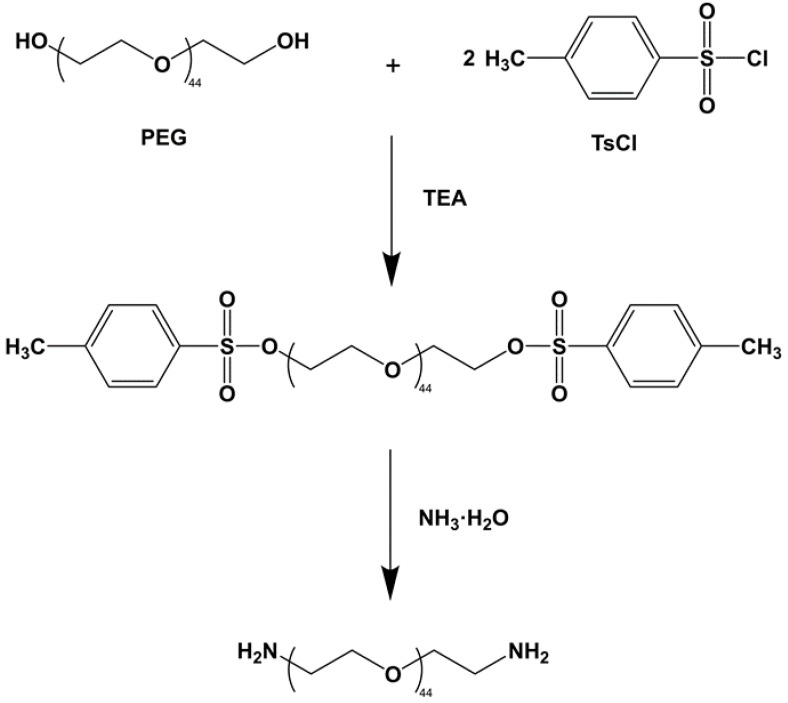
The synthesis of polyoxyethylene diamine.

TsO-PEG-OTs and ammonia water were added to a 150 mL autoclave and sealed at 140 °C for 6 h, then cooled to room temperature, and the aqueous phase was extracted with dichloromethane; sodium hydroxide aqueous solution (1M) was added, and the mixture was left standing after stirring for 4 h. The organic phases were combined, washed with deionized water to pH = 7, and freeze-dried to obtain a white powder product (H_2_N-PEG-NH_2_). The reaction process is shown in Figure 1.

### 2.3. Synthesis of Poly(amide-imide)—Polyethylene Glycol Copolymer (PAI-PEG)

BPDA and L-phenylalanine were uniformly dispersed in glacial acetic acid to form a homogeneous solution. The reaction system was stirred at room temperature (25 °C) for 12 h. Then, in an oil bath the temperature was raised to 110 °C, and the reaction was refluxed for 5 h. After the reaction was completed, vacuum distillation was performed to distill off the reaction solvent. The distillation temperature was 63 °C and the flow rate was 1 to 2 drops per second. Finally, the reaction product was washed 3 times with 1 mol/L hydrochloric acid aqueous solution. Suction filtration and vacuum drying at 30 °C for 12 h were performed to obtain L-phenylalanine-based imide diacid monomer [13].

**Figure 2:**
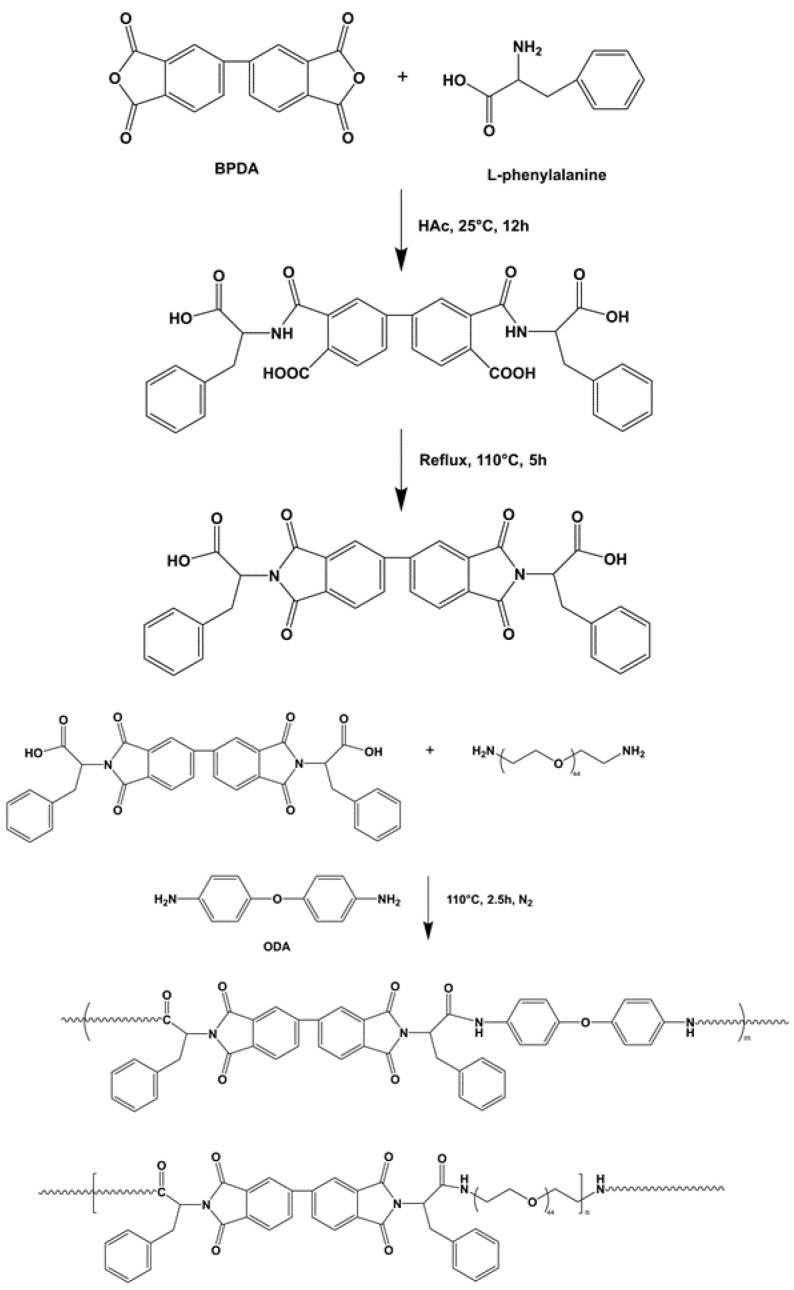
The synthesis of Poly(amide-imide)-polyethylene glycol copolymer(PAI-PEG).

ODA, polyoxyethylene diamine, and L-phenylalanine diamino acid monomer were added to molten tetrabutylammonium bromide to form a homogeneous solution, and TPP was added dropwise. The reaction system was placed in an oil bath at 110 °C and stirred for 2.5 h under the protection of N_2_. After the reaction was completed, anhydrous ethanol was added dropwise to the reaction solution, and the precipitated product was stirred, washed three times with anhydrous ethanol, filtered with suction, and dried under vacuum at 60 °C for 12 h to obtain the copolymer containing L-phenylalanine poly(amide-imide)—PEG block in the main chain (PAI-PEG). The reaction process is shown in Figure 2. Different samples’ names and compositions are shown in Table 1 and the ingredients are shown in Table 2.

### 2.4. Characterization of S1–S4

#### 2.4.1. Fourier Transform Infrared Spectrum Analysis (FTIR)

An infrared spectrometer (Nicolet 6700, Thermo Electron Scientific Instruments, Madison, WI, USA) was used to analyze the structure of PEG, TsO-PEG-OTs, H_2_N-PEG-NH_2_, and samples S1 to S4. The KBr tablet was used and the measurement range was 4000–400 cm^−1^.

#### 2.4.2. ^1^H NMR Analysis

A nuclear magnetic resonance spectrometer (Bruker Avance500, Bruker, Karlsruhe, Germany) was used to detect the hydrogen atom environment of synthetic polymers. Tetramethylsilane (Si(OH_3_)_4_) was used as the internal standard, and DMSO-d6 was used as the solvent.

#### 2.4.3. Hydrophilic Performance Analysis

The hydrophilic property of the polymer surface was detected by a water contact angle measuring instrument (JC2000C 50Hz, KRUSS, Hamburg, Germany). A point was measured every 5 mm. Each polymer was measured at least three times: points, multiple measurements, and average.

#### 2.4.4. Thermal Analysis

The thermal analysis (STA449F3/STA449F3, NETZSCH, Hamburg, Germany) was used to analyze the thermal properties of synthetic polymers. Nitrogen was used as the protective gas, the heating rate was 10 °C/min, and the test temperature range was 25 °C to 1000 °C.

#### 2.4.5. Mechanical Testing

The mechanical properties of the sample splines were tested according to GB/T1040-2006 “Plastic tensile test method”. Dumbbell-shaped test specimens with molded product specifications of 4 × 35 mm were tested for tensile properties and elongation at break using a universal material testing machine (M350/500, LLOYD, British). The speed was 1 mm/min. At least 5 samples were tested in each group.

To measure the recovery rate, a sample with a width of 4 mm and a length of L_0_ = 25 mm was used with a tensile speed of 50 mm/min. The tensile length was based on the stress-strain curve before the yield point. In this experiment, it was taken as 10 mm. Then, we waited for the sample to recover and measured the length as L. We used the following formula to determine the elastic recovery rate of the sample: X = (2L_0_ − L)/L_0_ × 100%.

#### 2.4.6. In Vitro Degradation Experiment

The degradation of S1–S4 of PAI-PEG was performed in vitro, mainly by immersing the material in PBS for 28 days. Samples at different time points were weighed, and the pH of the degradation solution and residual mass were measured. The surface morphology of the soaking material was observed with an electron microscope (SEM) (JSM IT200, JEOL, Akishima, Japan). A test sample with a regular shape was prepared, and the mass–volume ratio of the sample to the PBS was 1:20. The degradation device was placed in a 37 °C constant-temperature shaking box. Three samples were taken from each group on days 1, 3, 5, 7, 14, 21, and 28, and dried in a blast drying oven at 37 °C to constant weight.

#### 2.4.7. In Vitro Biocompatibility Assay

The in vitro biocompatibility of S1, S2, S3, and S4 was characterized through the observation of cell adhesion, proliferation, and differentiation. BMSCs were seeded onto the specimens with densities of 5 × 10^4^ cell/mL, 2 × 10^4^ cell/mL and 2.5 × 10^4^ cell/mL, for cell adhesion, proliferation, and differentiation, respectively [18,19]. After the BMSCs were cultured normally for 24 h, the cells adhered to the wall and were replaced with S1–S4 extract and the culture continued for 5 days. Then, 100 μL live/dead staining working solution was added to each well. After 10 min, images were observed and taken under a fluorescence microscope. S1–S4 samples were co-cultured with BMSCs for 3 days, and the cell morphology was observed by a confocal laser scanning microscope (CLSM) (FV1000, Olympus, Tokyo, Japan). The cell proliferation rate was assessed using the CCK-8 method. At 1, 3, and 5 days the formazan product was quantified by a microplate reader (Multiskan GO, Thermo SCIENTIFIC, Waltham, MA, USA).

## 3. Results and Discussion

### 3.1. Structure Characterization of PAI-PEG

**Figure 3:**
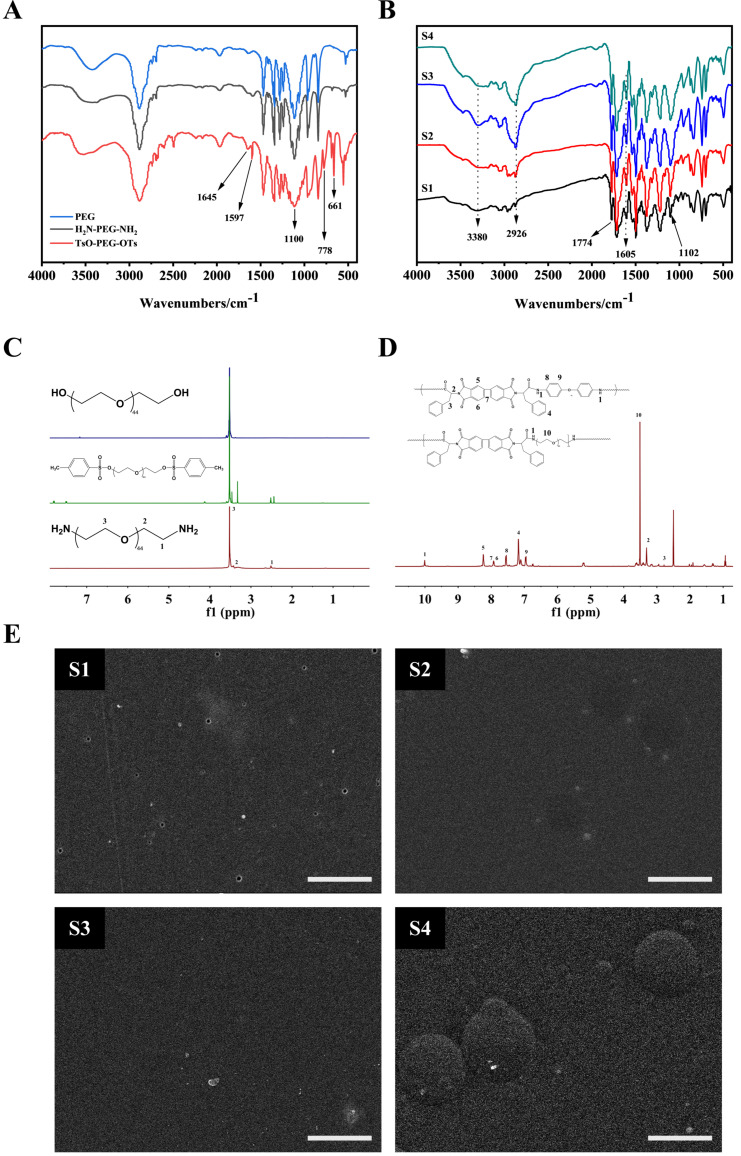
Structural characterization. (**A**): FTIR spectrum of PEG, TsO-PEG-OTs and H_2_N-PEG-NH_2_; (**B**): FTIR of S1–S4; (**C**): ^1^H NMR spectrum of PEG, TsO-PEG-OTs, and H_2_N-PEG-NH_2_; (**D**): ^1^H NMR spectrum of S1–S4; (**E**): S1–S4 surface SEM image. Scale bar = 500 μm.

Figure 3A shows the FTIR spectrum of PEG, polyethylene glycol p-toluene sulfonates (TsO-PEG-OTs), and double-terminal amino polyethylene glycols (H_2_N-PEG-NH_2_). Symmetrical stretching vibration, deformation vibration, and in-plane swing vibration of C-H were observed at 2877 cm^−^^1^, 1487 cm^−^^1^, and 802 cm^−^^1^ in the spectra of TsO-PEG-OTs and H_2_N-PEG-NH_2_. The characteristic C-O-C peak appears at 1100 cm^−^^1^. This peak was the main characteristic peak of PEG-Ots. The bond stretching peak, 1645 cm^−^^1^, was the characteristic peak of N-H of -NH_2_. FTIR analysis results showed that the synthesized structures TsO-PEG-OTs and H_2_N-PEG-NH_2_ were consistent with the expected structure. 

Figure 3B shows the FTIR spectrum of the copolymers (S1, S2, S3, and S4) of block poly(amido-imide)-polyethylene glycols with different contents of polyethylene glycol. It could be seen from the figure that 2933 cm^−^^1^ was an asymmetric stretching vibration of -CH_2_-. As the amount of polyethylene glycol increased, the absorption peak strength also increased; 1774 and 1716 cm^−^^1^ were symmetrical stretching vibrations of C=O on the imide ring; 1374 cm^−^^1^ was the stretching vibration of C-N on the imide ring; 1533 and 3380 cm^−^^1^ were the bending vibration and stretching vibration of N-H on amide group; 1223 cm^−^^1^ was the stretching vibration of C-N in amide group. The stretching vibration of C-N on the imide ring was 1394 cm^−^^1^; 842 cm^−^^1^ and 742 cm^−^^1^ were the characteristic peaks of benzene rings, and 1102 cm^−^^1^ was the characteristic peak of benzene ring ether. The characteristic peak of the bond was also the characteristic peak of the polyethylene glycol ether bond. The characteristic peak intensity increased with the increase in polyethylene glycol. FTIR spectrum results confirmed the successful preparation of block poly(amido-imide)-polyethylene glycol copolymers with different levels of polyethylene glycol.

As shown in Figure 3C, δ H (ppm): 3.52 was the absorption peak of the repeating segment (-CH_2_CH_2_O-); δ H (ppm): 2.51 and 3.50 peaks 1 and 2 were the amino-linked fluorene and β carbon Proton absorption peak. Combined with infrared spectroscopy analysis, we could see that the terminal hydroxyl group of PEG had been converted to the amino group of H_2_N-PEG-NH_2_. 

In Figure 3D, the proton peak at 9.996 ppm was the N-H proton on the amide group, which proved that the polymer skeleton contained the amide group. The peak at 5.198 ppm corresponded to the proton on the chiral carbon atom where L-phenyl propionic acid was connected to BPDA. The peak -CH-, and the H proton on the aromatic ring were in 5 different chemical environments. At position 5, due to the π–π conjugate effect between two adjacent benzene rings and the π–π conjugate effect of the carbon–oxygen double bond on the benzene ring and the imide ring, the chemical shift value moved to a low field, and the chemical shift value was high. However, because the π–π conjugate effect between the benzene rings was greater than the π–π conjugate effect of the carbon–oxygen double bond on the benzene ring and the imide ring, the density of the electron cloud around position 7 was slightly lower than position 6. Therefore, the chemical shift value of 7H on the benzene ring was higher than 6H. Similarly, the electron conjugation effect of p–π formed by the N atom and the benzene ring was lower than that of the O atom, so the density of the electron cloud at position 8 on the benzene ring was lower than that around position 9, and the chemistry displacement value of 8H on benzene ring was higher than 9H. The proton characteristic peak of polyethylene glycol-CH_2_CH_2_O- was at 3.51. Combined with the analysis of the infrared spectrum, it could be concluded that the block poly(amide imide)-polyethylene glycol copolymer was successfully prepared.

### 3.2. Thermal Analysis

**Figure 4:**
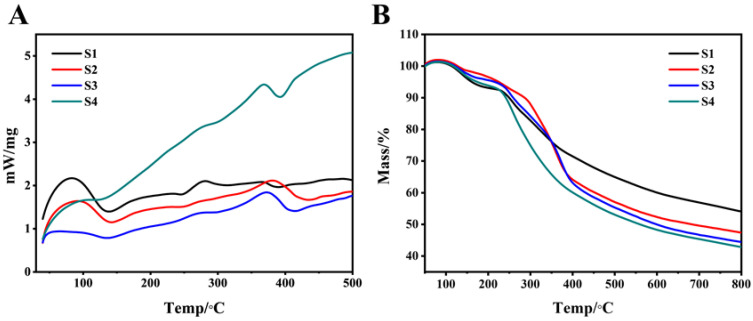
S1–S4 comprehensive thermal analysis spectrum. (**A**): S1–S4 DSC curve; (**B**): S1–S4 Tg curve.

Figure 4A shows the DSC curve of copolymer S1–S4. The crystallization temperatures (Tc) of S1–S4 were 138.3 °C, 136.8 °C, 130 °C, and 118 °C. With the increase in PEG content, the Tc of the copolymer moved to a lower temperature direction. This showed that as the soft segment increased, the ability of the copolymer to aggregate and crystallize decreased. We speculated that this phenomenon might be related to the following factors. First, it could be seen from Figure 3E that there was a certain degree of phase separation between PEG and PAI. The introduction of the random phase separation destroyed the chain structure of PAI and PEG, respectively, making the originally regular structure disordered, which affected the crystal growth of the respective segments, resulting in smaller grains or imperfect crystals. Second, with the increase in the content of the soft segment, the hard segment molecular chain would be restricted by the soft segment molecular chain, which made it difficult to crystallize after forming hydrogen bonds between amide groups. When the PEG content increased from 10% to 15%, these effects changed dramatically, as shown in Figure 4A; DSC curves of S3 and S4 showed great differences.

It can be seen from Figure 4B that at 200 °C, the Tg curves of all the groups began to drop. The weight loss rate of the S4 group had always been the highest, while the weight loss rate of the S1 group was the lowest, with S2 and S3 in between. A second thermal decomposition occurred at 450 °C. At this time, the weight loss rate of S4 was 48.7%, and the weight loss rate of S1 was 28.8%. This may indicate that as the content of the hard segment increased, the thermal decomposition of the soft segment was delayed, and the higher the content of the hard segment, the more PAI tended to agglomerate and lose less weight at the same temperature. Table 3 showed the weightlessness of the samples at different temperatures.

### 3.3. In Vitro Degradation Analysis

**Figure 5:**
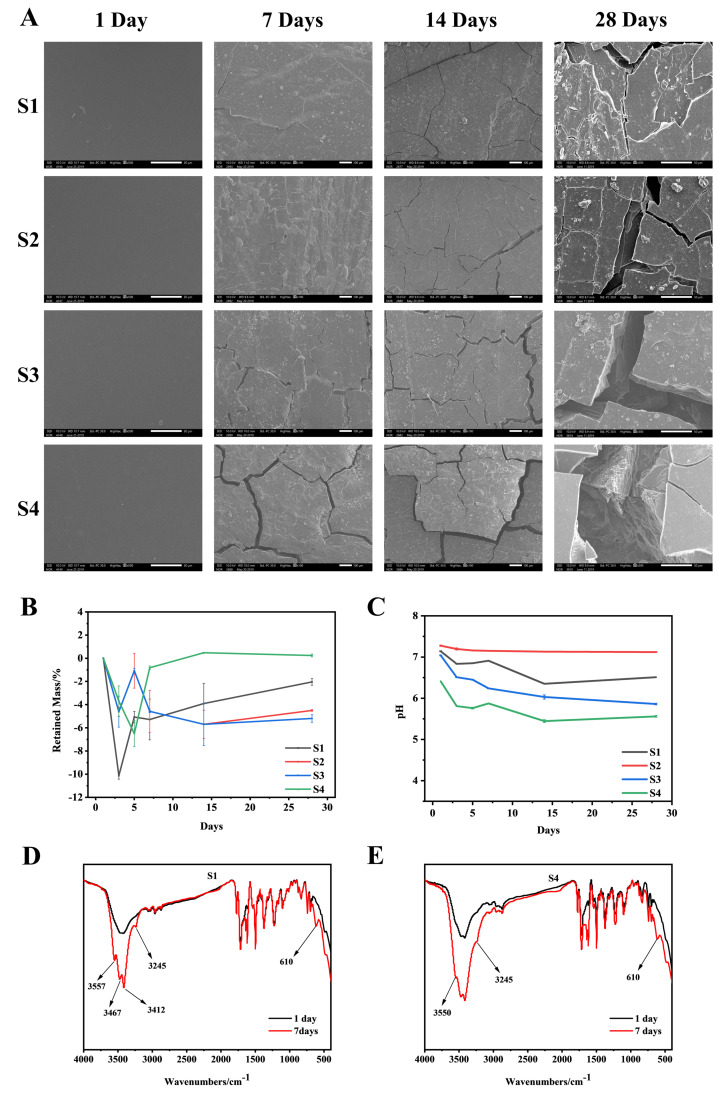
In vitro degradation experiment. (**A**): SEM surface morphology of S1–S4 degraded in PBS at 37 °C for 1, 7, 14, and 28 days. (**B**): residual mass during S1–S4 degradation; (**C**): during S1–S4 degradation pH value of degradation solution; (**D**): Infrared characterization chart of S1 without degradation and degradation for 7 days; (**E**): Infrared characterization chart of S4 without degradation and degradation for 7 days.

Figure 5A shows the surface morphology changes of S1–S4 after degradation for 1, 7, 14, and 28 days. After degradation for 7 days, cracks occurred on the surface of S1–S4. The cracks became larger with the higher PEG content. Large cracks appeared on the surface of S4 after 28 days of degradation, and the trend of increasing cracks with the increase of PEG content was consistent at 7, 14, and 28 days, which indicated that the surface morphology of degraded material was strongly related to the PEG content. The degradation data shows (Figure 5B) that the weight loss rate of the material in the initial stage of degradation displayed a decreasing trend, which means that the mass of the material did not decrease at the initial stage of degradation, but increased, and tended to degrade normally in the later stage of degradation, which may be related to the material’s adsorption of crystal water in PBS. As shown in Figure 5D,E, in the infrared characterization of the material after 7 days of degradation, compared with the undegraded material, a characteristic peak of crystal water appeared. This may explain why the weight loss rate of the material decreased during the initial stage of degradation, and the quality of degradation was less than the mass of crystal water adsorbed by the material. However, the content of PEG is not the only factor affecting the degradation rate of the copolymer. We speculated that when the amount of PEG was small, the hard segment PAI could tightly wrap the soft segment, slowing down the degradation rate of the copolymer. With the increase in PEG content, a large number of PEG chain segments were exposed to the degradation liquid environment, which increased the overall degradation rate of the copolymer.

The experimental data shows that the pH value of the degradation solution of different samples decreased with the increase in degradation time (Figure 5C), and the change rate of pH was different with the increase in PEG content, which was related to PEG degradation products. In the presence of enzyme-catalyzed alcohol dehydrogenase, PEG is metabolized to carboxylic acid, diacid and hydroxy acid metabolites by oxidation of its alcohol group [20]. However, the pH of the degradation solution could be maintained between 7.0 and 7.2 which was conducive to cell growth when the amount of 5 wt% of the material was added [21,22].

### 3.4. Mechanical Performance Analysis

**Figure 6:**
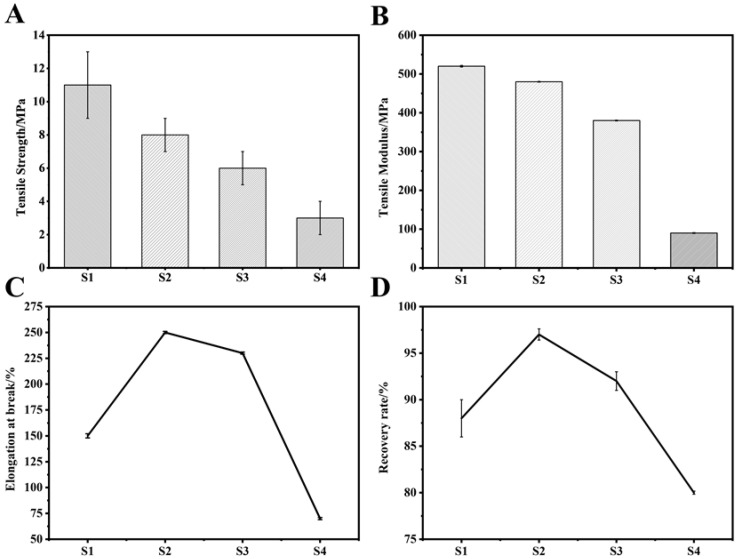
Analysis of mechanical properties of S1–S2; (**A**) Elastic strength; (**B**) Elastic modulus; (**C**) Elongation at break; (**D**) Recovery rate.

Figure 6A,B reflects the tensile strength and tensile modulus of the material. With the increase of the PEG content, the tensile strength and tensile modulus of the copolymer both showed a downward trend. This might be because the addition of PEG destroyed the PAI segment arrangement and reduced its crystallinity. PAI mainly provided mechanical strength for the copolymer, and PEG mainly provided its toughness. Therefore, when PEG was added, the tensile strength and tensile modulus decreased, but the elongation at break and recovery rate increased first and then decreased with the addition of PEG, reaching the maximum value in the S2 material group (252% and 97.5%, respectively, Figure 6B,D), while the values of the S1 material group were only 146% and 86%, respectively. When the material was stretched, physical cross-linking points would be formed between the segments. The cross-linking point could recover. Therefore, when the external force was removed, the material had recovery performance; when the amount of PEG added was small, the regularity of the crystal region was less damaged, and the corresponding existing physical cross-linking point had higher recovery rate. However, when the amount of added PEG increased, the crystallinity was severely damaged, the physical cross-linking points were reduced, and the recoverability that would otherwise have been provided naturally decreased with it. This was why the material group reached the maximum value in the S2 group and then showed a downward trend.

### 3.5. Hydrophilic Performance Analysis

**Figure 7:**
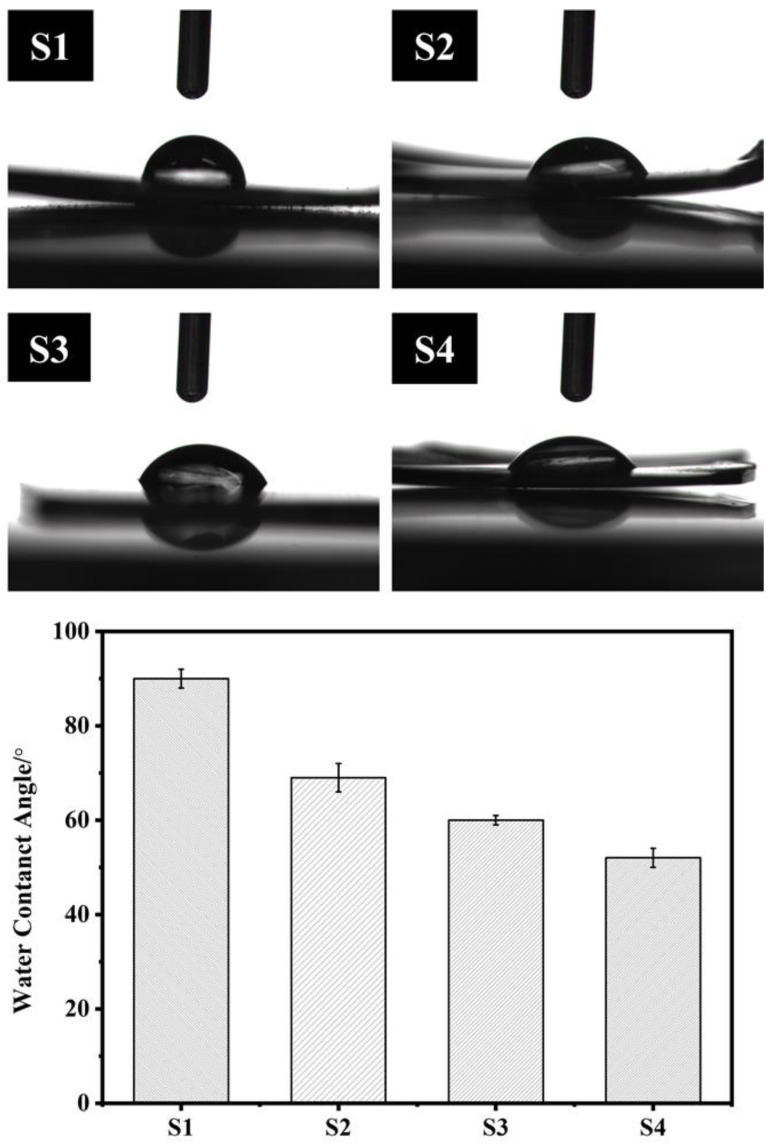
The water contact angle of the S1–S4.

As shown in Figure 7, after the introduction of PEG into the polymer, the contact angle of the polymer surface decreased from 90° to 52°, indicating that the introduction of PEG effectively improved the hydrophilic properties of the material surface. Compared with the degradable polymers PLLA (85°) and PLGA (74.5°) [22], the PAI-PEG copolymer had a better hydrophilic property. This result might be attributed to the fact that in an aqueous medium, the molecular conformation of PEG will change, and the ether bond (R-O-R) in the hydrophilic polyether chain segment is distributed on the chain surface, making it easy to combine with water around the polymer [14].

### 3.6. In Vitro Biocompatibility Analysis

**Figure 8:**
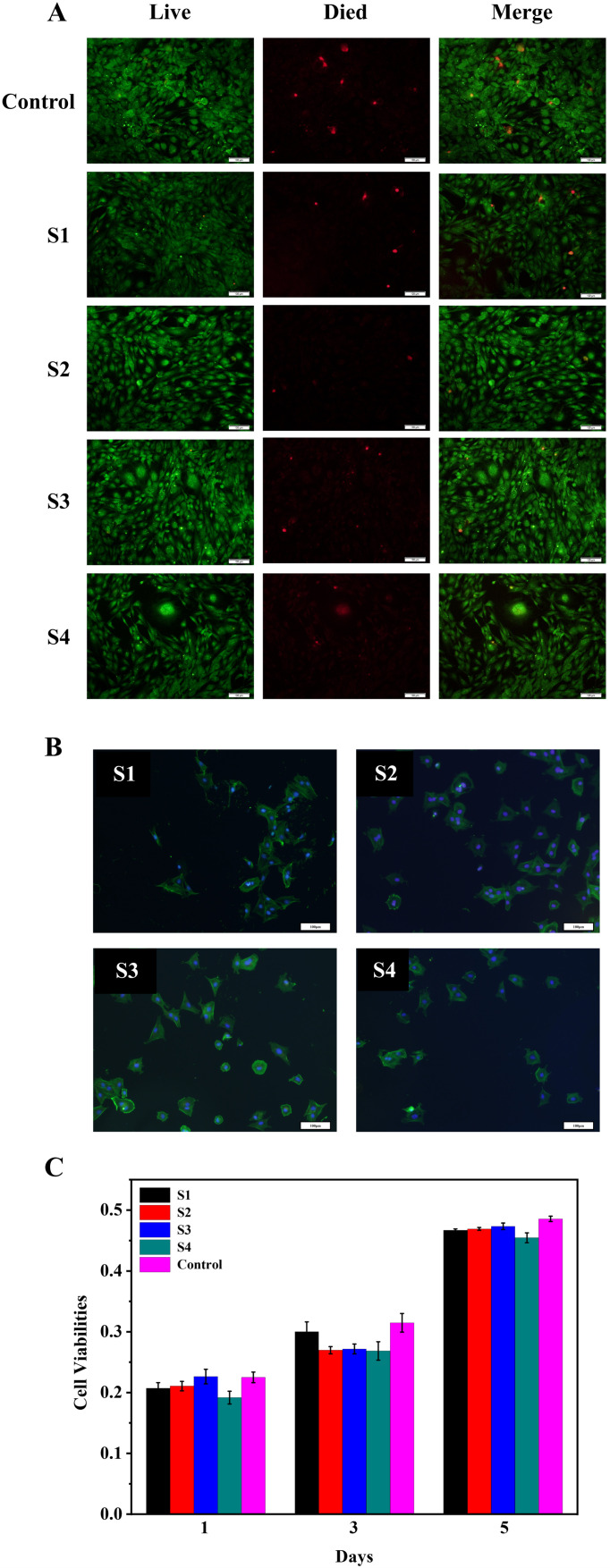
(**A**): Live/dead staining of the BMSCs that were cultured for 5 days on the S1–S4, respectively. (**B**): CLSM micrographs of the BMSCs adhesive on S1–S4 for 3 days. (**C**): Cell viabilities of the BMSCs that were cultured on the S1–S4 for 1, 3, and 5 days, respectively.

Figure 8A shows the live/dead staining of the BMSCs that were cultured for 5 days on the S1–S4. It could be seen from the figure that there was no significant difference in the results of live/dead staining in all material groups compared with the control group, but it could be seen that S2 had the best live/dead cell staining. Studies have shown that the growth rate of cells depends on the adhesion strength of cells on the surface of the material [23,24]. Figure 8B is a cell adhesion map of S1–S4 and BMSCs cells co-cultured for 3 days. From the figure, it could be seen that in all the material groups, the cells stretched almost in the shape of a pseudo-foot on the surface of the material, which indicated that the copolymer could provide a biocompatible surface environment for cell adhesion and growth and good cell adhesion. Figure 8C is a graph of cell viability after co-culture of S1–S4 and BMSCs. On the first day of co-cultivation, there was no significant difference in the absorbance between the material group and the control group; on the third day, there was no significant difference between the absorbance of S1 and the control group, and the absorbance of the other groups were lower than that of the control group. There was no significant difference in absorbance between the material group and the control group on the fifth day. This indicated that all material groups could promote the growth and proliferation of BMSCs. The results of in vitro biocompatibility tests showed that S1–S4 could promote the adhesion and proliferation of BMSCs in vitro. 

## 4. Conclusions

The main purpose of this study was to prepare a bone repair material with good mechanical properties and biocompatibility. The PAI-PEG copolymer was successfully prepared by solution polycondensation. The results showed that the S2 material exhibited the most excellent physical and chemical properties. In vitro biocompatibility of the S1–S4 showed that all material groups were non-toxic and could support the proliferation and adhesion of BMSCs. The S2 could potentially be used as bone repair implants in orthopedic surgery in the future.

## Figures and Tables

**Table 1:** Different samples’ names and compositions.

Specimens’ Names	Compositions
S1	PAI-PEG-0
S2	PAI-PEG-5
S3	PAI-PEG-10
S4	PAI-PEG-15

**Table 2:** The synthesis of PAI-PEG composites with different amounts of PEG.

Sample	wt% ^a^	PEG2000 (g)	Imide Diacid Monomer (g)	ODA (g)
S1	0	0	5.97	1.9624
S2	5	1	5.97	1.9
S3	10	2	5.97	1.8
S4	15	3	5.97	1.7

^a^ represents the mass percentage of PEG added.

**Table 3:** The weightlessness of the samples at different temperatures.

	Mass/% (T = 200/℃)	Mass/% (T = 500/℃)
S1	7.5	28.8
S2	1.8	37.4
S3	5.63	40.4
S4	8.38	48.7

## Data Availability

Not applicable.
